# Emergence of *Usutu virus*, an African Mosquito-Borne *Flavivirus* of the Japanese Encephalitis Virus Group, Central Europe

**DOI:** 10.3201/eid0807.020094

**Published:** 2002-07

**Authors:** Herbert Weissenböck, Jolanta Kolodziejek, Angelika Url, Helga Lussy, Barbara Rebel-Bauder, Norbert Nowotny

**Affiliations:** * Institute of Pathology and Forensic Veterinary Medicine, Vienna, Austria; † Institute of Virology, University of Veterinary Medicine, Vienna, Austria; ‡ United Arab Emirates University, Al Ain, United Arab Emirates

**Keywords:** *Usutu virus*, *Flavivirus*, avian mortality, Austria, pathology, virology

## Abstract

During late summer 2001 in Austria, a series of deaths in several species of birds occurred, similar to the beginning of the *West Nile virus* (WNV) epidemic in the United States. We necropsied the dead birds and examined them by various methods; pathologic and immunohistologic investigations suggested a WNV infection. Subsequently, the virus was isolated, identified, partially sequenced, and subjected to phylogenetic analysis. The isolates exhibited 97% identity to *Usutu virus* (USUV), a mosquito-borne *Flavivirus* of the Japanese encephalitis virus group; USUV has never previously been observed outside Africa nor associated with fatal disease in animals or humans. If established in central Europe, this virus may have considerable effects on avian populations; whether USUV has the potential to cause severe human disease is unknown.

*Usutu virus* (USUV) is a relatively unknown member of the mosquito-borne cluster within the *Flavivirus* genus, closely related to important human pathogens such as *Japanese encephalitis virus* (JEV), *Murray Valley encephalitis virus* (MVEV), *Dengue virus* (DENV), *Yellow fever virus* (YFV), *Saint Louis encephalitis virus* (SLEV), and *West Nile virus* (WNV) (1–3). Isolated for the first time from mosquitoes in South Africa in 1959 and named after a river in Swaziland (4), USUV was sporadically isolated from several mosquito and bird species over the next decades (5–7). Only two isolations have been reported from mammals, one from *Praomys* sp. (African soft-furred rats) and one from a man with fever and rash (5). The virus has never been associated with severe or fatal diseases in animals or humans, and it has never before been observed outside tropical and subtropical Africa.

From the beginning of August through mid-September 2001, a considerable die-off of Eurasian Blackbirds (*Turdus merula*) was observed in and around Vienna, Austria. Some observers reported obviously sick blackbirds, which showed signs of apathy and ruffled plumage. Within 5 days in mid-August, five Great Gray Owls (*Strix nebulosa*) died in the Tiergarten Schönbrunn Vienna Zoo. In addition, many dead Barn Swallows (*Hirundo rustica*) were observed in the Austrian federal state of Upper Austria, 200 km west of Vienna. Investigating this episode of avian deaths in Austria, we determined USUV as the causative agent.

## Methods

In total, we received six blackbirds, five owls, and one swallow for investigation. At necropsy (estimated postmortem times between 24 h and 48 h), tissue samples were fixed in 7% buffered formalin. After embedding in paraffin wax, 4-µm sections were stained with hematoxylin and eosin.

Paraffin-embedded tissue samples were immunostained with a polyclonal mouse antibody to WNV (B. Murgue, Institut Pasteur, Paris) and a polyclonal rabbit antibody to *Tick-borne encephalitis virus* ([TBEV] strain Neudoerfl, H. Holzmann, Klinisches Institut für Virologie, Vienna) using the Avidin-Biotin Complex technique (8).

RNA was extracted from 140-µL organ homogenates or cell culture suspensions by using the QIAamp Viral RNA Mini Kit (Qiagen GmbH, Hilden, Germany). After aligning available nucleotide (nt) sequences of various mosquito-borne flaviviruses and determining highly conserved genomic regions, we designed three pairs of oligonucleotide primers (to amplify a wide range of mosquito-borne flaviviruses) and used them in the reverse transcription-polymerase chain reaction (RT-PCR) assays: 5´-TACAACATGATGGGVAARAGAGAGA-3´ (nt position 9031–9055 of WNV GenBank accession no. NC 001563) and 5´-AGCATGTCTTCYGTBGTCATCCAYT-3´ (nt position 10115–10091), resulting in a 1,084-bp amplification product; 5´-GARTGGATGACVACRGAAGACATGCT-3´ (nt position 10090–10115) and 5´-GGGGTCTCCTCTAACCTCTAGTCCTT-3´ (nt position 10832–10807), amplifying a 743-bp PCR product; and 5´-GCCACCGGAAGTTGAGTAGA-3´ (nt position 10460–10479 of WNV no. NC 001563) and 5´-GCTGGTTGTGCAGAGCAGAA-3´ (nt position 10908–10889), resulting in a 449-bp amplicon. Reverse transcription and amplifications were performed in a continuous RT-PCR method by using the QIAGEN OneStep RT-PCR Kit (Qiagen GmbH). Each 25-µL reaction mixture contained 5 μL 5X buffer (final MgCl_2_ concentration 2.5 mM), 0.4 mM deoxynucleoside triphosphate (dNTP), 10 U recombinant RNasin Ribonuclease Inhibitor (Promega, Madison, WI), 40 pmol forward and reverse primers, 1 µL enzyme mix, and 2.5 µL template RNA. Reverse transcription was performed for 30 min at 50°C. Following an initial denaturation for 15 min at 95°C, the reaction mixture was subjected to 45 cycles of heat denaturation at 94°C for 30 s, primer annealing at 60°C for 30 s, and DNA extension at 72°C for 1 min, completed by a final extension of 10 min at 72°C. Following RT-PCR, we performed electrophoresis on 20 µL of the amplicons in a 1.2% Tris acetate-EDTA-agarose gel. The gel was stained with ethidium bromide, and the bands were observed under UV light.

We excised the fragments from gel, extracted DNA, and performed sequencing PCR. The PCR products were sequenced in both directions by using the ABI Prism 310 genetic analyzer automated sequencing system (Perkin Elmer Instruments, Wellesley, MA). The nucleotide sequences were compiled and aligned with the corresponding sequences deposited in the GenBank database. Finally, we constructed a phylogenetic tree based on a 1,035-nt fragment in the NS5 genomic region. The following sequences have been included in the phylogenetic analysis: AF013384, *Koutango virus*; AF013413, *Yaounde virus*; D00246, Kunjin virus; AF202541, WNV (New York 1999); M12294, WNV; AF013367, *Cacipacore virus*; U15763, JEV; AF013360, Alfuy virus; AF013389, MVEV; AF013412, USUV (South Africa); AF452643, USUV (Austria); AF013416, SLEV; M93130, DENV (type 3); and AF013417, YFV. The phylogenetic analysis was carried out by using Phylogeny Interference Program Package (PHYLIP), version 3.57c (available from: URL: http://evolution.genetics.washington.edu/phylip.html). We generated bootstrap resampling analysis of 100 replicates with the SEQBOOT program. Distance matrices were generated with the DNADIST/Neighbor-Joining program; a translation/transversion ratio of 2.0. AF013417 (YFV) was used as outgroup.

Paraffin-embedded tissue samples were processed as described (9). For detection of WNV nucleic acid, an antisense digoxigenin-labeled riboprobe complementary to nt 4966–5439 of WNV strain NY1999 was generated from plasmid pWNNY-88B-14 (W.I. Lipkin, Emerging Diseases Laboratory, Irvine, CA). The final concentration of the probe was approximately 0.5 ng/µL. For detection of USUV nucleic acid, we used a digoxigenin-labeled oligonucleotide probe with the sequence: 5´-TCGCATAACTTTCACCACCTTGTGTTTGTAGGTCAGCTC-3´, which is complementary to nt 367-328 of the accessible partial sequence of the NS5 gene of USUV (GenBank accession no. AF013412). The final probe concentration was approximately 0.25 ng/µL.

## Results

**Figure 1:**
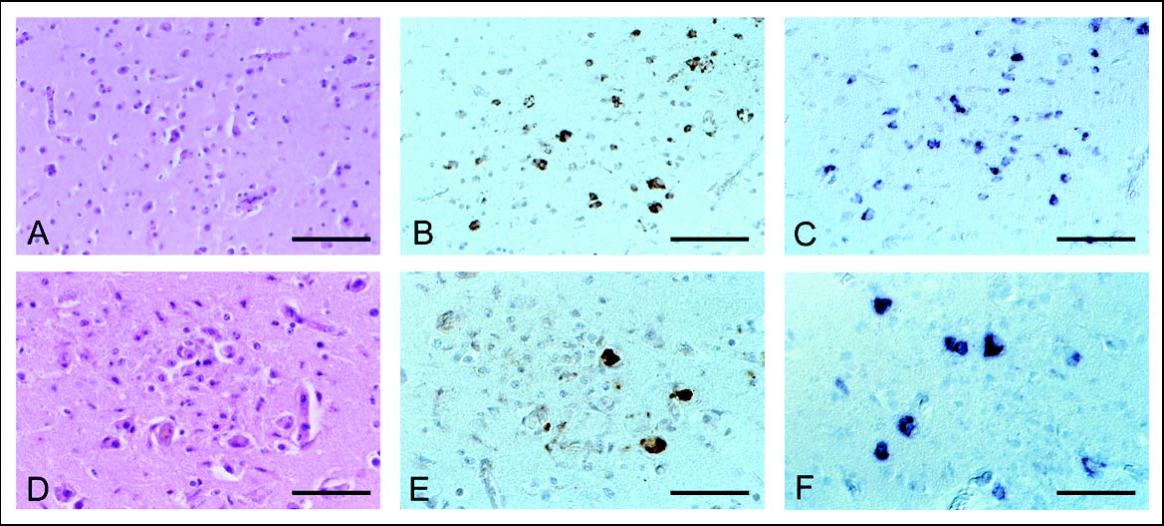
Histology and detection of viral signals in paraffin-embedded tissue sections of birds infected with *Usutu virus* (USUV). A–C, Eurasian Blackbird; D–F, Great Gray Owl; A, no histologic lesions present, hematoxylin and eosin staining; B immunohistochemistry, using a polyclonal antibody to *West Nile virus*, shows numerous positive neurons; C, in situ hybridization with USUV-specific oligonucleotide probe shows a staining pattern comparable with that in B; D, microglial nodule within the cerebral cortex, hematoxylin and eosin staining; E, immunohistochemistry shows single positive neurons within a glial nodule; F, in situ hybridization shows several positive neurons next to a glial nodule. Original magnification, x 130 (A–C), x 200 (D–F).

Necropsy showed grossly swollen livers and spleens, as well as seromucous enteritis in all blackbirds and owls; histology showed various degrees of multifocal acute necrosis in liver and spleen. Although the blackbirds did not show obvious histologic brain lesions, the owls had encephalitis, predominantly shown as multifocal areas of neuronophagia and microgliosis (Figure 1, A and D). Pathologic and immunohistochemical investigation of the swallow did not yield useful results because of severe autolysis.

Immunohistochemistry (IHC) with polyclonal antibodies to WNV was positive in 10 of 11 brains, showing reaction products in neurons and their processes and in the cytoplasm of microglial cells in glial nodules (Figure 1, B and E). Positive reactions were also present in kidney, spleen, liver, lung, and autonomous ganglia of the gastrointestinal tract. Brain and kidney samples from WNV-infected birds from the United States and Israel, respectively, were positive controls; blackbirds that died from trauma were negative controls. IHC with polyclonal antibodies to TBEV, another flavivirus found in central Europe, showed negative results. RT-PCR with WNV-specific primers and ISH with a WNV-specific probe were negative. Infection with a flavivirus related to WNV would account for the cross-reactivity of a polyclonal antibody used in IHC and the negative outcome of the WNV-specific assays.

Organ homogenates of blackbirds and owls were added to Vero cell cultures. After 24–48 hours, a cytopathic effect of cell rounding could be observed; 1 to 2 days later, the affected cells detached and floated in the medium.

RT-PCRs with universal flavivirus primers resulted in clear PCR amplification products of the expected lengths. The primers were designed to amplify overlapping PCR products in the NS5 genomic region of mosquito-borne flaviviruses. RT-PCRs were performed both on the original organ homogenates and on cell culture suspensions, with identical results. Despite a poor state of preservation, organ homogenates of the swallow also proved positive by RT-PCR.

**Figure 2:**
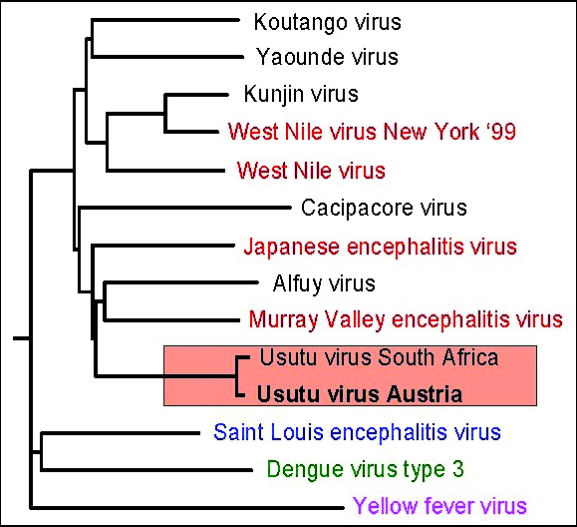
Phylogenetic analysis of several members of the *Japanese encephalitis virus* (JEV) group and selected other mosquito-borne flaviviruses demonstrates the close genetic relationship of the Austrian *Usutu virus* (USUV) isolate with the South African USUV (red underlay); well-known members of the JEV group are highlighted in red; distinct branches are formed by *Saint Louis encephalitis virus*, *Dengue virus* (type 3), and *Yellow fever virus*. The partial nucleotide sequence of the Austrian USUV isolate used in the phylogenetic tree has been deposited in the GenBank database under accession no. AF452643.

The PCR products (1,084 bp, 743 bp, and 449 bp) were directly sequenced in both directions; the compiled nucleotide sequences (a stretch of 1,877 bp, representing approximately 17% of flavivirus genome) were aligned and compared with other sequences by using the Basic Local Alignment Search Tool (BLAST) search (National Center for Biotechnology Information, National Institutes of Health, Bethesda, MD). The sequence obtained from the Austrian dead birds was 97% identical with a 1,035-nt fragment of USUV (GenBank accession no. AF013412; [2]), an African mosquito-borne flavivirus in the JEV group. To investigate the genetic relationship of the Austrian USUV with other flaviviruses in the JEV antigenic complex, we constructed a phylogenetic tree by using the programs in the PHYLIP package; we also included in the phylogenetic analysis three other important mosquito-borne flaviviruses (YFV, SLEV, and DENV3). The phylogenetic tree (Figure 2) demonstrates the close genetic relationship between USUVs isolated in South Africa and in Austria; therefore, we classified the Austrian USUV as part of the JEV group of flaviviruses. At the amino-acid level, the Austrian and the South African USUV isolates proved (in the investigated 1,035-nt region) to be 100% identical.

With an oligonucleotide probe specific for USUV, ISH showed presence of viral nucleic acid in the cytoplasm of neurons in a distribution pattern closely matching IHC (Figure 1, C and F). Regarding other organs, however, kidneys of only two birds were positive, probably reflecting RNA degradation due to postmortem times >24 h.

## Discussion

We demonstrated the presence of a mosquito-borne flavivirus, never before observed outside tropical and subtropical Africa, in the continental climate of central Europe, where winter temperatures are as low as –20°C. Since we also detected USUV nucleic acid in a Barn Swallow, the virus was probably introduced to the Austrian bird population by swallows or other migrating birds. Bird die-offs in various bird species in different areas of Austria suggest that the virus has already adapted to local mosquito species, which are probably transmitting the virus. Isolating the virus from local mosquitoes has not been attempted thus far but is planned. During a retrospective survey of paraffin-embedded blackbird tissues by IHC and RT-PCR, we also detected USUV in a blackbird that died a year earlier (2000). A partial nucleotide sequence of this USUV proved to be 100% identical to the sequences of the 2001 USUV isolates. Although no severe bird die-offs were observed in 2000, we think that USUV may already be established and be overwintering in Austria (rather than being newly introduced in 2 consecutive years). Comparable with the introduction of WNV to North America in 1999, where the virus propagated in local mosquito species and then rapidly spread from New York to >20 states in the United States and Canada (10), we foresee a similar scenario for USUV in Europe.

This study shows for the first time that USUV is highly pathogenic for several different species of birds. Because full sequence data of USUV isolates are not available (only a short [1,035-bp] fragment of one USUV isolate has been deposited in the GenBank database), we cannot fully compare the strain isolated here with the African isolates. We cannot provide information on amino acid changes that might contribute to altered pathogenic properties. For the closely related WNV, the pathogenicity for birds seems to depend on the virus strain and whether the virus affects a previously exposed or unexposed population. The WNV strain that appeared in North American birds in 1999 is closely related to a strain isolated in Israel; this strain is associated with avian deaths in both countries (11–14). Certain other WNV strains, such as those responsible for recent outbreaks in Romania and Russia in humans (15,16) and in Italy and France in equines (17,18), were not associated with avian deaths. Also, the fact that certain avian species, such as Eurasian Blackbirds, Great Gray Owls, and Barn Swallows in Austria, are especially vulnerable to USUV infection, is reminiscent of the observation that WNV in North America has primarily affected American Crows and Blue Jays (19,20).

 The emergence of WNV in the United States in 1999 and USUV in central Europe in 2000–2001 is an indication of future virus activity. The next mosquito-borne flavivirus, which might be introduced to regions far from its original habitats, may be highly pathogenic for humans, farm animals, or pets, as many strains of JEV group are. We could speculate whether global warming or other environmental factors may have contributed to the introduction and maintenance of USUV, formerly restricted to tropical and subtropical areas, in a much colder climate. As a consequence of the introduction of USUV to Central Europe, surveillance programs for mosquito-borne flaviviruses in general (based on virus detection in mosquitoes and dead birds, as well as epidemiologic investigations) should be established in Europe, like those initiated in the United States after the first occurrence of WNV (19,20). Moreover, we will have to fully sequence USUV, establish serologic test systems, and evaluate the spread and pathogenic potential to control this new virus infection in Central Europe.
